# Male gender predicts mortality in a large cohort of patients receiving antiretroviral therapy in Uganda

**DOI:** 10.1186/1758-2652-14-52

**Published:** 2011-11-03

**Authors:** Edward J Mills, Celestin Bakanda, Josephine Birungi, Keith Chan, Robert S Hogg, Nathan Ford, Jean B Nachega, Curtis L Cooper

**Affiliations:** 1 Faculty of Health Sciences, University of Ottawa, Ottawa, Canada; 2 British Columbia Centre for Excellence in HIV/AIDS, Vancouver, Canada; 3 The AIDS Support Organization (TASO), Headquarters, Kampala, Uganda; 4 Centre for Infectious Disease Epidemiology and Research, University of Cape Town, South Africa; 5 Centre for Infectious Diseases, Stellenbosch University, Stellenbosch, South Africa; 6 Johns Hopkins Bloomberg School of Public Health, Baltimore, MD, USA; 7 Division of Infectious Diseases, The Ottawa Hospital, Ottawa, Canada

## Abstract

**Background:**

Because men in Africa are less likely to access HIV/AIDS care than women, we aimed to determine if men have differing outcomes from women across a nationally representative sample of adult patients receiving combination antiretroviral therapy in Uganda.

**Methods:**

We estimated survival distributions for adult male and female patients using Kaplan-Meier, and constructed multivariable regressions to model associations of baseline variables with mortality. We assessed person-years of life lost up to age 55 by sex. To minimize the impact of patient attrition, we assumed a weighted 30% mortality rate among those lost to follow up.

**Results:**

We included data from 22,315 adults receiving antiretroviral therapy. At baseline, men tended to be older, had lower CD4 baseline values, more advanced disease, had pulmonary tuberculosis and had received less treatment follow up (all at p < 0.001). Loss to follow up differed between men and women (7.5 versus 5.9%, p < 0.001). Over the period of study, men had a significantly increased risk of death compared with female patients (adjusted hazard ratio 1.43, 95% CI 1.31-1.57, p < 0.001). The crude mortality rate for males differed importantly from females (43.9, 95% CI 40.7-47.0/1000 person-years versus 26.9, 95% CI 25.4-28.5/1000 person years, p < 0.001). The probability of survival was 91.2% among males and 94.1% among females at 12 months. Person-years of life lost was lower for females than males (689.7 versus 995.9 per 1000 person-years, respectively).

**Conclusions:**

In order to maximize the benefits of antiretroviral therapy, treatment programmes need to be gender sensitive to the specific needs of both women and men. Particular efforts are needed to enroll men earlier into care.

## Background

Although the global prevalence of HIV among women has remained stable at 50%, in sub-Saharan Africa, it is markedly higher in females than in males [1]. Similarly in Africa, young women have a higher incidence of HIV infection [1-3]. As a focused public health strategy, efforts to improve access to treatment, research and human rights for women and girls have recently received particularly special attention. Substantially less attention has been focused on men [4].

Scale up in access to care and treatment has been comparatively more successful for women than for men [5,6]. Recently, there has been growing recognition that men face important challenges for both access to care and delivery of care [4,7-9]. Men may be more difficult to provide care for due to issues of occupation, residence or cultural beliefs. Reasons for health-seeking behaviour differences between men and women are poorly understood. Cultural norms that label sickness as a sign of weakness for men have fostered a reluctance of care seeking among men [2]. Men's actions, and those of women, may be constrained by tradition and influenced by cultural beliefs and social norms [10].

In addition, employment-related migration keeps men away from their partners and families for prolonged periods and contributes to increased vulnerability by predisposing them to high-risk behaviours [10]. These risk factors make men both vulnerable to HIV infection and discouraged from seeking testing and treatment. In Africa, antenatal care services act as a common entry point to HIV testing, treatment and care services for women, as pregnant women are routinely tested for HIV. No such opportunity exists for men [4,8].

In this paper, we report on outcomes and life expectancy of men and women receiving combination antiretroviral therapy (cART) in a nationally representative cohort in Uganda.

## Methods

### Participants

Our analysis includes routinely collected data on all patients aged 14 and older who initiated antiretroviral therapy at clinics run by The AIDS Support Organization (TASO) in Uganda. This cohort has been described in detail elsewhere [11].

Founded in 1987, TASO provides psychosocial support, clinical care and cART to individuals infected with HIV. TASO began providing widespread cART in 2004 and now provides cART to more than 24,000 patients. Since its inception, TASO has expanded the scope of its work to include nutritional support, vocational training, HIV education and capacity building of healthcare workers. TASO's mission is to restore hope and improve the quality of life of individuals, families and communities affected by HIV and related diseases. TASO provides support to more than 100,000 HIV-infected patients and supports family members through education, counselling and educational stipends. TASO provides services through 11 regional TASO centres and 35 mini-TASO centres in rural districts.

TASO receives its core cART funding through the United States President's Emergency Plan for AIDS Relief (PEPFAR), as well as from Irish AID and Swedish SIDA. The Ugandan Ministry of Health refers newly diagnosed HIV-infected children and adults to TASO clinical sites for care. Many of the populations serviced by TASO represent marginalized and neglected groups, including infants and children, orphans, conflict-affected populations, internally displaced people, widows, prisoners and family members of HIV-infected patients that may require urgent support. TASO programmes emphasize adherence and retention, and include innovative approaches to maintaining patient interest, including drama and social groups, diary writing and involvement of patients in clinical duties to become "expert patients".

TASO provides a range of services, including HIV testing, clinical care, provision of cART and psychosocial support. Laboratory services are limited, but include CD4 analysis, complete blood analysis, tuberculosis and malaria diagnosis, and urine assessments. Criteria used for initiation of cART at TASO include World Health Organization (WHO) Stage III or IV or a CD4 cell count below 250 cells/mm^3^. Criteria for children's clinical admittance into the TASO cART programme are based on Ugandan Ministry of Health Guidelines. Children are eligible for cART if they have WHO paediatric Stage III, advanced Stage II, or Stage I with CD4 cell percentage (%) < 15% for those over 18 months of age, and < 20% for those under 18 months of age [12]. The Uganda Ministry of Health National Antiretroviral Treatment and Care Guidelines for Adults and Children have not yet been updated to reflect WHO's newest recommendations for clinical staging and immunological classification [13].

When a patient attends a TASO clinic, clinicians will complete standardized patient forms detailing patient demographics, as well as clinical, psychosocial and drug utilization data. These data are then hand entered, in duplicate, into the TASO clinical administrative data collection. Patients are provided with a unique confidential identifying number. Patients requiring cART typically receive an initiation regimen based on a non-nucleoside reverse transcriptase inhibitor with first-line treatment comprising nevirapine, lamivudine and stavudine and boosted lopinavir, didanosine and zidovudine as second-line treatment [14]. Patients requiring treatment for TB co-infection will receive their combination care at a TASO clinic.

Patients aged 14 years and older who initiated antiretroviral therapy at TASO clinics in Uganda between 1 January 2004 and 1 January 2010 were included in this study. These patients were followed until either the time of death or the end of the study period (1 January 2010). For this analysis, we extracted the following information: age at the start of the antiretroviral therapy; gender; baseline CD4 count; WHO clinical disease stage; presence of tuberculosis or sexually transmitted infections at treatment initiation; date when they were last seen; and, where applicable, date of all-cause mortality, non-disease mortality, or defaulting from care (defined as a three-month untraceable absence from a clinic).

### Analysis

Patient characteristics by gender are described using medians and interquartile ranges for continuous variables and counts and percentages for categorical data and compared using a chi-squared test. Survival distributions for male and female patients are estimated using the Kaplan-Meier method and compared by log-rank test. Survival was calculated from the start date of antiretroviral therapy to the date of death. Patients who were lost to follow up were censored at the date when they were last seen. Patients who were still alive at the date when the study ended were censored at this date. Survival times were expressed in months. We applied a weighted analysis whereby 30% of patients lost to follow up were assumed to be dead, weighted by baseline CD4 and age, as suggested by Egger *et al *[15,16]. To account for missing baseline CD4 cell counts, we conducted our analyses using the multiple imputation method of SAS [17].

Potential years of life lost (PYLL) before age 55 were used to examine the effect of HIV on premature mortality. PYLL represent the sum of years that individuals lost because of premature mortality. PYLL are a convenient summary measure that account for not only the number of deaths, but also the ages at which death occurs. To obtain PYLL, we grouped deaths according to age at death by five-year increment categories. The total number deaths for a particular cause in each five-year age group are multiplied by the average number of years remaining in that age group to age 55 years, as follows:

$$\text{PYLL }=\Sigma di\left(55-Yi\right)$$

where, *Y_i _*is the age at the midpoint of age group *i*.

Survival times were expressed in months. Unadjusted and adjusted Cox proportional hazards regression was conducted in order to quantify the effect of gender on survival, adjusting for age, CD4 status and WHO clinical disease stage. This analysis included point and confidence interval estimates for the hazard ratios of death for each factor. Hazard proportionality was assessed by analysis of scaled Schoenfeld residuals. All significance tests were two-sided with a p value of < 0.05 considered significant. All analyses were conducted using SAS version 8 (SAS Institute, Cary, NC). Additional file 1 presents results for the overall analysis based on only documented deaths.

### Institutional approval

Approval to conduct this study was received from the administrative headquarters and Research Ethics Board of TASO Uganda, an approved Ugandan National Science and Technology Ethics Review Board, and the Research Ethics Boards of the University of British Columbia and the University of Ottawa. Because this analysis was based on routine clinical data, retrospective individual patient consent was not required.

## Results

**Figure 1:**
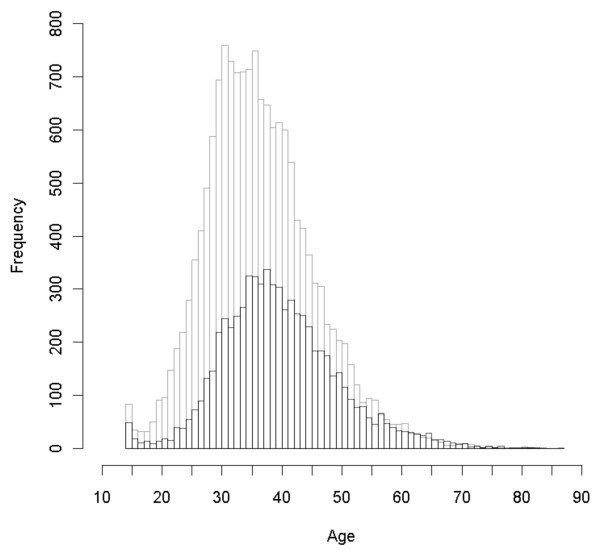
**Distribution of included patients by age and sex**. Grey shading, female; black shading, male.

We included data from 22,315 adults receiving antiretroviral therapy, representing 59,436 person-years. The majority (19,885; 89%) were aged between 14 and 49 years and 2430 (11%) were aged 50 years or older. Figure 1 displays the distribution of patients by age and sex. Baseline CD4 cell count, WHO stage at initiation, presence of tuberculosis and duration of follow up all differed significantly between male and female patients (Table 1). Over the course of the study, 918 females were lost to follow up (5.9%) and 515 males (7.5%) (p < 0.001). Patients lost to follow up had a lower median CD4 than those not lost (105 interquartile range 73-207 vs. 144, interquartile range 34-181, p < 0.001).

**Table 1:** Characteristics of included patients at baseline

Characteristics	Category	Total	Female n (%)	Male n (%)	p value
Age	14-19	333	233 (1.5)	100 (1.5)	< 0.001
	20-29	3486	2866 (18.5)	620 (9.1)	
	30-39	9774	6966 (45)	2808 (41.2)	
	40-49	6292	4035 (26)	2257 (33.1)	
	50+	2430	1392 (9)	1038 (15.2)	
	Total (n)	22,315	15,492	6823	

CD4	< 50	3452	2173 (16.9)	1279 (22.8)	< 0.001
	50-99	2942	1944 (15.1)	998 (17.8)	
	100-149	3410	2391 (18.5)	1019 (18.2)	
	150-249	5740	4152 (32.2)	1588 (28.3)	
	250+	2954	2233 (17.3)	721 (12.9)	
	Total (n)	18,498	12,893	5605	

WHO Stage at antiretroviral therapy initiation	Stage 1	465	339 (3.4)	126 (2.7)	< 0.001
	Stage 2	7985	5615 (56)	2370 (51.2)	
	Stage 3	4982	3294 (32.9)	1688 (36.5)	
	Stage 4	1220	778 (7.8)	442 (9.6)	
	Total (n)	14,652	10,026	4626	

TB at antiretroviral therapy initiation	No	21,207	14,847 (95.8)	6360 (93.2)	< 0.001
	Yes	1108	645 (4.2)	463 (6.8)	
	Total (n)	22,315	15492	6823	

Sexually transmitted infection diagnosed at antiretroviral therapy initiation	No	17,634	11,579 (74.7)	6055 (88.7)	< 0.001
	Yes	4681	3913 (25.3)	768 (11.3)	
	Total (n)	22,315	15,492	6823	

Switch from first antiretroviral therapy	No	20,675	14,313 (92.4)	6362 (93.2)	0.024
	Yes	1640	1179 (7.6)	461 (6.8)	
	Total (n)	22,315	15,492	6823	

Median months of follow-up time (interquartile range)	-	-	32 (20-47)	30 (18-39)	< 0.001

**Figure 2:**
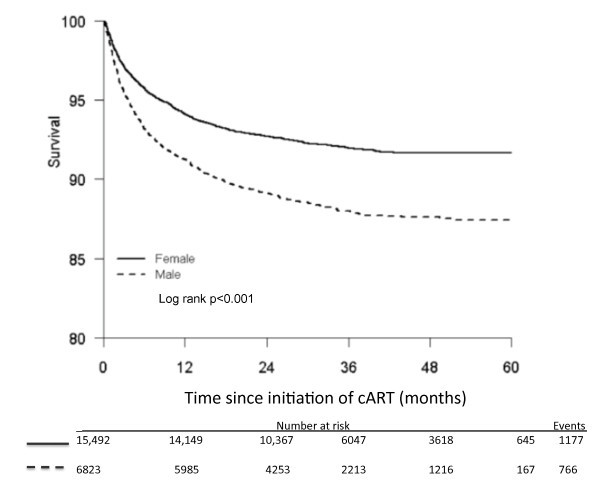
**Kaplan-Meier survival curves on time to death**.

There were 1498 deaths that were accounted for in our analysis. We imputed a further 445 deaths from lost-to-follow-up patients. Additional file 1 provides the results without adjusting for loss to follow up. Overall, men had a significantly increased risk of death compared with female patients (hazard ratio 1.53, 95% confidence interval [CI] 1.40-1.68, p < 0.001). Most deaths (n = 740) occurred within the first three months of initiating antiretroviral therapy (302 in men and 438 in women). There was no difference in non-disease mortality by gender (odds ratio, 0.95, 95% CI 0.59-1.54, p = 0.86). The probability of survival among males compared with females was 95.5% (95% CI 95.0-96.0) and 97.2 (95% CI 96.9-97.4%) at three months; 93.2% (95% CI 92.6-93.8%) and 95.8% (95% CI 95.5-96.1%) at six months; 91.2% (95% CI 90.6-91.9%) and 94.1% (95% CI 93.7-94.5%) at 12 months; and 89.1% (95% CI 88.4-89.9%) and 92.7% (95% CI 92.3-93.1%), respectively, at 24 months (Figure 2).

### Univariate and multivariable Cox proportional hazards regression

Table 2 presents the unadjusted and adjusted hazard ratios (HRs) for each variable. We found that gender remained independently associated with mortality after adjusting for other factors (adjusted HR 1.43, 95% CI 1.31-1.57). We additionally found that lower CD4 status at baseline was associated with mortality.

**Table 2:** Proportional hazards regression for time to death

Variable	Unadjusted hazard ratio (95% CI)	p value	Adjusted hazard ratio (95% CI)	p value
Gender (male versus female)	1.53 (1.40-1.68)	<0.001	1.43 (1.31-1.57)	<0.001
Age				
14-19	1.00		1.00	
20-29	0.98 (0.67-1.42)	0.895	1.05 (0.72-1.53)	0.794
30-39	0.90 (0.62-1.29)	0.566	0.96 (0.67-1.39)	0.835
40-49	0.87 (0.60-1.26)	0.459	0.93 (0.65-1.35)	0.713
50+	1.20 (0.82-1.75)	0.338	1.31 (0.90-1.91)	0.164
CD4 at antiretroviral therapy initiation (per 100 cells/mm^3^)	0.65 (0.61-0.68)	<0.001		
CD4 count				
<50	1.00		1.00	
50-99	0.83 (0.73-0.94)	0.005	0.82 (0.72-0.94)	<0.001
100-149	0.63 (0.55-0.72)	<0.001	0.63 (0.55-0.73)	<0.001
150-249	0.44 (0.39-0.50)	<0.001	0.45 (0.39-0.51)	<0.001
250+	0.37 (0.31-0.45)	<0.001	0.38 (0.32-0.46)	<0.001

World Health Organization Stage at ART initiation				
Stage 1	1.00			
Stage 2	0.99 (0.67-1.47)	0.957		
Stage 3	2.04 (1.38-3.03)	<0.001		
Stage 4	3.78 (2.52-5.67)	<0.001		

The crude mortality rate was 31.8 (95% CI 30.3-33.2) per 1000 person-years for the overall cohort. Females had a lower crude mortality rate compared with males: 26.9 (95% CI 25.4-28.5) per 1000 person-years versus 43.9 (95% CI 40.7-47.0) per 1000 person-years, respectively. Potential years of life lost (PYLL) was 795.0 per 1000 person-years for the overall cohort. Similarly, PYLL was lower for females than males (689.7 versus 995.9 per 1000 person-years, respectively).

## Discussion

Our study demonstrates that male HIV patients in Uganda have consistently worse survival outcomes compared with their female counterparts. Our study findings build upon evidence suggesting that male outcomes are consistently worse in Africa [5,18-20]. Given the low coverage of antiretroviral therapy among men in Uganda and other parts of Africa, an emphasis on involving men in HIV testing and the route to treatment is critical if we are serious about addressing the vulnerability of women for HIV acquisition.

Public health planning can frequently be counter intuitive [21]. In the context of global developmental programming, the concepts of "gender and development" and "women and development" have been frequently constructed as one and the same [22], and increasing calls for consideration of gender relations in the AIDS response have been mostly met with a focus on girls and women. However, the specific vulnerabilities of boys and men in this new configuration of gender relations are rarely addressed. Indeed, very little is known about how to engage men with directed efforts to change their risk-taking and health-seeking behaviours.

It is likely that much of the successes of engaging women in clinical care and their more positive outcomes stem from directed efforts to target women, as well as logistic efforts to access women at a health-seeking moment, such as in antenatal clinics. Efforts to engage men at antenatal services have had poor outcomes. It seems more likely that efforts aimed at their places of work or aimed at peer educators may have better success [8].

Circumcision clinics are gaining prominence in much of Africa as an intervention to reduce male infections [23]. This may provide an important opportunity to test men for their serostatus and direct them into care. Retention in programmes will be a major challenge as men frequently travel for work, and therefore targeted and novel interventions to maintain or increase retention in care is also badly needed [8].

Several studies have found that males fare worse than females in terms of cART access and outcomes [5,18-20,24]. While this variable has typically been included as a covariate in regression analysis, it is infrequently examined in detail. A paper from South Africa looked at a small cohort of 2196 patients receiving cART, 33% of whom were male [19]. As with our study, men presented at a later age and with more advanced disease. Two studies from Malawi examined mortality outcomes between males and females with a very high crude mortality rate (123.2/1000 person years) and found a heightened mortality among males regardless of clinical features (HR 1.90, 95% CI 1.57-2.29) as well as increased loss to follow up (HR 1.66, 95% CI 1.43-1.92) [20,25]. A study involving more than 12,000 patients in Tanzania found an increase in mortality among men (HR 1.19, 95% CI 1.05-1.30), immunologic non-response defined as a CD4 cell count of less than 100 cells/mm^3 ^after at least six months of cART (HR 1.74, 95% CI 1.44-2.11) and loss to follow up (HR 1.19, 95% CI 1.10-1.30) than that in women [6]. The largest evaluation until now, examining 11,153 patients across four countries with close clinical monitoring, found an HR of 1.17 (95% CI 1.02-1.35) for male mortality after adjustments for other expected covariates [18]. A previous analysis using this cohort examined life expectancy and found dramatic differences between sexes: males at the age of 20 could expect to live an additional 19.1 years while females could expect 30.6 years [26].

Strengths of our study include the large and nationally representative sample involving patients across many age groups. As TASO engages active retention of defaulting patients, we have minimized loss to follow up compared with other AIDS service organizations that frequently experience about 40% losses [27]. Attrition typically occurs at two distinct times, pre- and post-antiretroviral therapy initiation [28]. A TASO study found that approximately 26% of 637 patients eligible for antiretroviral therapy did not initiate treatment, and most often, these were males [16]. We applied a weighted analysis to adjust for an attrition effect on mortality [15].

Our study represents just one country. A previous systematic review, of early experiences providing antiretroviral therapy, found a ratio of 2.3:1 of women receiving antiretroviral therapy compared with men across 13 countries in sub-Saharan Africa [29]. A further limitation is that we do not have data on viral failure and cannot be sure if treatment failures are associated with gender, although a large study of better resourced clinics found results similar to ours [18]. Finally, as an observational study, we recognize that our estimates are subject to potential confounding by unmeasured variables. Other studies have found that level of education and pregnancy status at initiation influence mortality, and may partially explain our mortality estimates [5,30,31].

## Conclusions

In conclusion, our study has important implications for future generations of individuals infected and affected by HIV/AIDS. Funding agencies should recognize that males and females are necessary components of most households, and plan for their interventions appropriately.

## Competing interests

The authors declare that they have no competing interests.

## Authors' contributions

EM, CB, JB and KC conceived the study design, analyzed and interpreted the data and wrote the manuscript. EM, CB and JB contributed to the design of the study and revised the manuscript. KC, RH, NF, JN and CC participated in the analysis and interpretation of the data and revised the manuscript. All of the authors approved the final version of the manuscript submitted for publication.

## Supplementary Material

Additional file 1 **Analysis according to unadjusted data**. Click here for file
