# Supplementary material for: Male gender predicts mortality in a large cohort of patients receiving antiretroviral therapy in Uganda
Source: J Int AIDS Soc. 2011 Nov 3;14:52. doi: 10.1186/1758-2652-14-52 (PMC3220631; doi:10.1186/1758-2652-14-52)

**Additional File 1.**

**Table A. Proportional hazards regression for time to death (using only documented deaths)**

| **Variable** | **Unadjusted hazard ratio (95% CI)** | **p value** | **Adjusted hazard ratio (95% CI)** | **p value** |
| --- | --- | --- | --- | --- |
| Gender (male vs. female) | 1.54 (1.39-1.71) | <0.001 | 1.42 (1.28-1.58) | <0.001 |
| Age  14-19  20-29  30-39  40-49  50+ | 1.00  0.81 (0.54-1.21)  0.82 (0.55-1.21)  0.78 (0.53-1.16)  1.06 (0.71-1.60) | 0.301  0.312  0.225  0.773 | 1.00  0.87 (0.58-1.31)  0.89 (0.60-1.31)  0.85 (0.57-1.27)  1.19 (0.79-1.78) | 0.516  0.542  0.436  0.413 |
| CD4 at ART initiation (per 100 cells/mm^3^) | 0.63 (0.59-0.67) | <0.001 |  |  |
| CD4 count  <50  50-99  100-149  150-249  250+ | 1.00  0.76 (0.66-0.88)  0.56 (0.48-0.66)  0.37 (0.32-0.43)  0.32 (0.26-0.40) | <0.001  <0.001  <0.001  <0.001 | 1.00  0.75 (0.65-0.87)  0.57 (0.48-0.66)  0.38 (0.33-0.44)  0.33 (0.27-0.41) | <0.001  <0.001  <0.001  <0.001 |
| WHO Stage at ART initiation  Stage 1  Stage 2  Stage 3  Stage 4 | 1.00  1.04 (0.65-1.68)  2.27 (1.41-3.63)  4.45 (2.74-7.23) | 0.862  <0.001  <0.001 |  |  |

**Table B. Probabilities of survival by gender (3, 6, 12, 24 months) (using only documented deaths)**

|  |  | **Probability of survival** | |  |
| --- | --- | --- | --- | --- |
| **Gender** | **No. at risk** | **3 month** | **Lower 95% CI** | **Upper 95% CI** |
| **Female** | 14854 | 0.978 | 0.976 | 0.980 |
| **Male** | 6390 | 0.963 | 0.958 | 0.967 |
|  |  | **Probability of survival** | |  |
| **Gender** | **No. at risk** | **6 month** | **Lower 95% CI** | **Upper 95% CI** |
| **Female** | 14552 | 0.966 | 0.963 | 0.969 |
| **Male** | 6178 | 0.944 | 0.939 | 0.950 |
|  |  | **Probability of survival** | |  |
| **Gender** | **No. at risk** | **12 month** | **Lower 95% CI** | **Upper 95% CI** |
| **Female** | 14145 | 0.953 | 0.95 | 0.957 |
| **Male** | 5984 | 0.93 | 0.924 | 0.936 |
|  |  | **Probability of survival** | |  |
| **Gender** | **No. at risk** | **24 month** | **Lower 95% CI** | **Upper 95% CI** |
| **Female** | 10366 | 0.943 | 0.939 | 0.947 |
| **Male** | 4252 | 0.915 | 0.908 | 0.922 |

**Table C. Crude death rates by gender (using only documented deaths)**

| **Gender** | **Rate (1000 y)** | **s.e.** | **Lower 95% CI** | **Upper 95% CI** |
| --- | --- | --- | --- | --- |
| **Female** | 21.3 | 0.7 | 20.0 | 22.7 |
| **Male** | 34.8 | 1.4 | 32.0 | 37.6 |
| **Overall** | 25.2 | 0.7 | 23.9 | 26.5 |

**Figure A. Kaplan-Meier survival curves on time to death (using only documented deaths)**


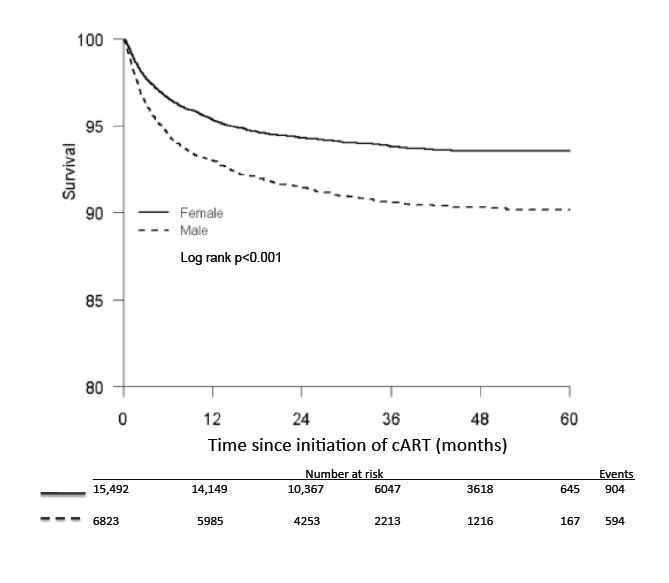

Supplement: Additional file 1 — Analysis according to unadjusted data. [file 1758-2652-14-52-S1.DOC]
